# Hemostasis and complement in allogeneic hematopoietic stem cell transplantation: clinical significance of two interactive systems

**DOI:** 10.1038/s41409-024-02362-8

**Published:** 2024-07-14

**Authors:** Dimitrios A. Tsakiris, Eleni Gavriilaki, Ioanna Chanou, Sara C. Meyer

**Affiliations:** 1 University of Basel, Basel, Switzerland; 2 MEDISYN SA, Lucerne, Switzerland; 3 Second Propedeutic Department of Internal Medicine, Aristotle University of Thessaloniki, Thessaloniki, Greece; 4 Department of Biomedical Sciences, School of Health Sciences, International Hellenic University, Thessaloniki, Greece; 5 Department of Hematology and Central Hematology Laboratory, Inselspital, Bern University Hospital, University of Bern, Bern, Switzerland

**Keywords:** Innate immunity, Leukaemia, Stem-cell therapies

## Abstract

Hematopoietic stem cell transplantation (HCT) represents a curative treatment option for certain malignant and nonmalignant hematological diseases. Conditioning regimens before HCT, the development of graft-versus-host disease (GVHD) in the allogeneic setting, and delayed immune reconstitution contribute to early and late complications by inducing tissue damage or humoral alterations. Hemostasis and/or the complement system are biological regulatory defense systems involving humoral and cellular reactions and are variably involved in these complications after allogeneic HCT. The hemostasis and complement systems have multiple interactions, which have been described both under physiological and pathological conditions. They share common tissue targets, such as the endothelium, which suggests interactions in the pathogenesis of several serious complications in the early or late phase after HCT. Complications in which both systems interfere with each other and thus contribute to disease pathogenesis include transplant-associated thrombotic microangiopathy (HSCT-TMA), sinusoidal obstruction syndrome/veno-occlusive disease (SOS/VOD), and GVHD. Here, we review the current knowledge on changes in hemostasis and complement after allogeneic HCT and how these changes may define clinical impact.

## Introduction

Hematopoietic stem cell transplantation (HCT) currently serves as a primary curative option for selected malignant and nonmalignant hematological diseases [1–3]. The conditioning regimens applied before HCT, the development of graft-versus-host disease (GVHD) in the allogeneic setting, and delayed immune reconstitution contribute to a wide array of early and late complications resulting from tissue or humoral damage [4]. Biological regulatory defense systems, encompassing humoral and cellular reactions such as hemostasis or the complement system, are variably involved in these complications. Traditionally described separately as distinct entities, their likely parallel evolutionary development [5], along with common targets shared in their defense reactions, associates them with each other [6–9]. The endothelium, which suffers from HCT complications, has emerged as a common target of both systems, placing them inevitably side by side whenever endothelial cells are damaged and inflammation occurs [7, 10, 11]. Here, we review the incidence, pathophysiology and management of complications after allogeneic HCT, which are related to hemostasis and/or complement system dysregulation, with a specific focus on how these complications interconnect and mediate major clinical impacts early and late after HCT.

## Hemostasis and complement as interconnected systems

**Fig. 1:**
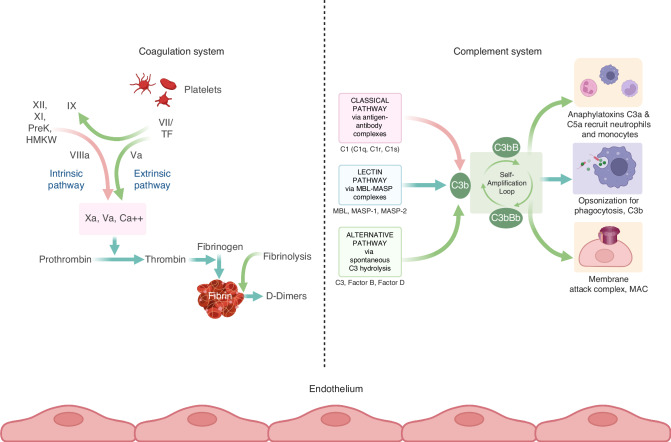
Simplified scheme of the coagulation (left panel) and complement (right panel) cascades active on a fictitious endothelial lesion. A dotted line separates the illustration of the two biological systems only for better understanding. The endothelial bed as a common ground upon which they develop, binds the systems functionally and can promote activation and bilateral interaction of both mechanisms, depending on the nature or the intensity of the triggering event. LEFT PANEL: Va, Xa, VII, IX, XI, XII: coagulation enzymes, PreK: precallikrein, TF: tissue factor, Ca + +: calcium ions, green arrows: activation of the extrinsic pathway of coagulation, red arrow: activation of the intrinsic pathway of coagulation, bluish arrows: activation of the common pathway. RIGHT PANEL: Activation of the complement system over three distinct pathways going over to a common amplification loop and thus triggering humoral and/or cellular innate immunity reactions [7, 16].

Hemostasis, described as a network of proteases and cellular components, aims to stop bleeding through clot formation [12, 13]. It involves primary mechanisms related to the vessel wall, endothelium, and platelets, as well as secondary activation of coagulation enzymes, leading to thrombin generation and clot formation. Both mechanisms require a ground of phospholipids, usually consisting of cell membranes (e.g. endothelium, extravascular tissue, or platelets), at the site of tissue injury upon which they interplay (Fig. 1).

Complement, a complex system of proteases, undergoes chain reactions to activate enzymes, aiming to destroy pathogenic cells through cell lysis. The complement system demonstrates self-regulatory capacity and is activated locally by regulatory enzymes, driving its action to a focused destruction of target cells [14–16]. Complement activation occurs through three main pathways, the classical pathway, the lectin pathway, and the alternative pathway, converging to a common final pathway where C3 convertase produces anaphylatoxins C3a and C5a, as well as the membrane attack complex (MAC). These products play a central role in the generation of inflammation, creating transmembrane channels that induce lysis and cell death in pathogenic cells. The complement system mainly exerts biological effects through cell lysis, the inflammatory response, pathogen opsonization, and immune complex removal (Fig. 1) [14–16].

**Fig. 2:**
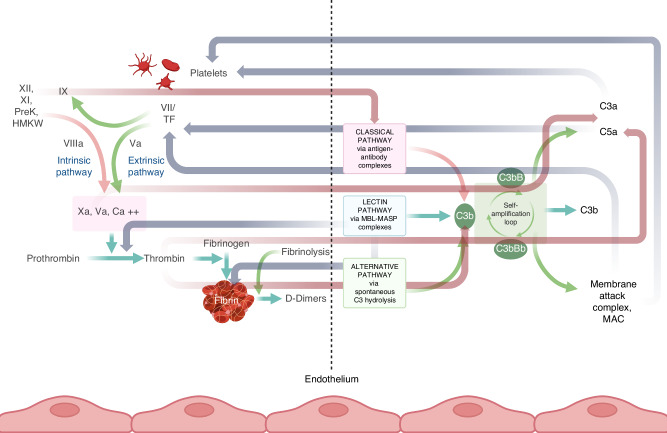
Simplified scheme of the coagulation (left panel) and complement (right panel) cascades on a fictitious common endothelial lesion. Horizontal arrows depict interactions of components of the two systems illustrating how hemostasis can activate complement and vice versa (Va, Xa, VII, IX, XI, XII: coagulation enzymes, PreK: precallikrein, TF: tissue factor, Ca + +: calcium ions, MASP-2: mannose-binding protein-associated serine protease-2, MAC: membrane attack complex, C3a-C3b-C5a: complement factors). LEFT PANNEL: Red arrows illustrate how components of the coagulation system can activate complement at various levels of the cascade implicating the impact of coagulation on innate immunity reactions. RIGHT PANNEL: Grey arrows illustrate how complement components can activate coagulation reactions at variable levels of the cascade linking inflammatory reactions to thrombotic events [7, 16].

It has been postulated that hemostasis and complement, as defense systems, share parallel evolutionary origins dating millions of years ago [16, 17]. While complement and hemostasis in their current evolutionary state seem to function independently (Fig. 1), numerous mutual interactions between them have been described under both normal and pathological conditions (Fig. 2) [7, 9, 18–20] and their interconnections have become evident in several settings:

I. *Immediate activation upon tissue damage*. One common feature of hemostasis and complement systems is their “front line” position in response to any type of tissue wound; these systems are integral components of the immediate reaction to tissue damage and crucial factors in natural immunity. The induction of a wound is promptly followed by a cascade of enzymatic reactions resulting in the formation of fibrin and the activation of the immune system to respond to the specific site of injury. Specifically, local inflammatory mechanisms triggered at the site of vascular injury are enhanced by factors from the hemostatic and complement systems. Both hemostasis and complement unfold on a bed of damaged cells, often the endothelium. Following an endothelial lesion, an immediate hemostatic reaction is to locally activate platelets. In addition to forming a platelet thrombus, activated platelets express p-selectin on their surface and release soluble *p*-selectin. *P*-selectin binds noncovalently to complement factor C3 activation fragments, thereby enhancing the mechanisms that cause local inflammation in vascular lesions [7, 21, 22]. Moreover, megakaryocytes and platelets were found to contain C3, which is released upon platelet activation at the site of local injury [23]. Conversely, interactions have also been described in which complement activation enhances primary hemostasis through the binding of C1q to von Willebrand factor, a major adhesion molecule at the site of tissue damage, which mediates platelet adhesion and aggregation [19].
II. *Common triggers and functional inhibitors*. Several studies have shown that the hemostatic and complement systems share common triggers and regulators and that they interact with each other on several levels. It has been shown in vivo in a murine model deficient in complement component C3 that C5a can be generated by proteolytic activation of C5 by thrombin, a central hemostasis enzyme [7]. Activated coagulation enzymes, such as FIXa, FXa, and FXIa, can proteolytically activate C3 and C5, thus triggering the common pathway of complement activation. Coagulation factor XIIa mediates the activation of C1, thus initiating the classical pathway of complement activation. Fibrinogen and fibrin can enhance the initiation of the lectin pathway on the surface of pathogens [24, 25]. Conversely, complement components can enhance hemostasis [9]. Activation products of the lectin pathway, such as MASP-2, can promote thrombin generation. C5a can induce the release of tissue factor from endothelial cells and neutrophils. C1q, an initiator of the classical pathway, can enhance primary hemostasis by interacting with von Willebrand factor, a major adhesion molecule between platelets and the endothelium [19].
III. *Interaction with neutrophil extracellular traps (NETs)*. The hemostasis and complement systems are known to interact with neutrophil extracellular traps (NETs), which function as major bacterial defense mechanisms [26, 27]. The process of NETosis is a form of neutrophil cell death, with NETs representing sticky ‘nets’ composed of modified chromatin from neutrophils harboring pieces of destroyed pathogens. NETs can function as a trigger for clot formation by carrying tissue factor and generating thrombin, depending on the stimulus that initiates them, but they can also activate coagulation enzymes by acting as negatively charged surfaces. Complement, on the other hand, may also induce NET formation or could be activated by existing NETs, thus forming an enhancing feedback loop with coagulation [26].
IV. *Disease states with simultaneous involvement of complement and hemostasis*. It is becoming increasingly evident that the hemostatic and complement systems functionally interact in multiple pathologic conditions with a major clinical impact. The functional intercalation and mutual enhancement of hemostatic and complement activation have been reported in transplant-associated thrombotic microangiopathy (HSCT-TMA) [28–30], sinusoidal obstruction syndrome/veno-occlusive disease (SOS/VOD) [31, 32] and graft-versus-host disease (GVHD) [30, 33, 34] in the posttransplantation setting. Similar interconnections have also been described independent of allogeneic HCT, such as paroxysmal nocturnal hemoglobinuria (PNH)[35, 36]^,^ atypical hemolytic uremic syndrome-induced microangiopathy (aHUS) [37, 38], C3 glomerulopathy [39, 40], autoimmune and alloimmune hemolytic anemia [41, 42] and antiphospholipid syndrome (APS) [43]. Overall, it is becoming clear that understanding the multiple interactions of the two systems provides novel insights, including insights into molecular targets, as a basis for new therapeutic interventions.

## Clinical impact of hemostasis and complement alterations on HCT

Hemostasis in the peri-transplant setting has been extensively studied since changes in hemostatic function are involved in several types of transplant complications. While early studies primarily referring to HCT with bone marrow as a hematopoietic cell source cannot be easily extrapolated to the current practice of HCT with peripheral blood-derived hematopoietic stem cells [44–46], recent studies have focused mostly on biomarkers of hemostasis and endothelial cell activation given their central involvement in serious complications, such as HSCT-TMA or GVHD [10, 11, 47–50].

### Macro-thrombotic events and HCT

In the early phase of HCT, bleeding events are the most common hemostatic complications, particularly when myeloablative conditioning regimens are used [51]. Overall, the incidence of clinically symptomatic bleeding in the peri-transplant period is estimated to be 15–27% [46, 50–52]. Thrombosis can occur early in the first weeks or late up to some years after HCT and involves various vascular regions [46, 50, 51, 53–56]. It has been reported with variable frequencies, ranging between 0.5 and 23.5% [57]. Zahid et al. [57] published a meta-analysis of 23 included studies published between 1993 and 2014 and reporting thrombotic events after HCT. The overall incidence of thromboembolic events, incl. deep vein thrombosis and pulmonary embolism in the first year after HCT was estimated to be approximately 5% (4–7%) with no differences between allogeneic and autologous setting. If acute GvHD was present the incidence increased to 47% and with chronic GvHD to 35% respectively. In other studies, the incidence of catheter-related thrombosis was found to be between 2.5 and 7.8% reported at one month or one year after HCT [52, 54, 58]. Arterial thrombosis was also reported with a cumulative incidence of 6% over 15 years [52]. Risk factors associated with thrombosis in HCT patients are prolonged hospitalization, cancer-related thrombotic risk, myeloablative conditioning, immunomodulatory agents, severe infections and active GvHD. However, the heterogeneity of the patient cohorts in the studies does not easily allow for a precise cross-comparison and analysis of the existing data [50, 57]. Overall, endothelial damage resulting in endothelial dysfunction represents a common feature in the context of GvHD. Features like intensive cell trafficking in periods of severe cytopenia and infections, as well as subsequent stress myelopoiesis contribute to imbalances in hemostatic function, leading to both bleeding and thrombosis [59–61].

Additional pathogenetic mechanisms associated to clonal hematologic diseases might emerge as contributing factors to thrombosis. Clonal hematopoiesis of indeterminate potential (CHIP) is a recognized entity in the evolution of hematologic malignancies [62]. Intriguingly, CHIP is associated with higher overall mortality and has been linked to the development of cardiovascular and/or thrombotic diseases, including myocardial infarction, cerebrovascular thrombosis, and deep vein thrombosis [63, 64]. A mechanistic explanation for these thrombotic events is not entirely clear yet, but they might be linked to mechanisms triggering local inflammation, driven by the respective clonal mutations. CHIP is age-related and becomes a relevant concern in the context of HCT, as candidate patients undergoing this procedure are increasingly older in recent decades [62, 65]. CHIP can manifest in three ways in patients receiving HCT: it may exist pre-transplant in clones that survive conditioning and the graft vs. leukemia effect, it may be acquired through donor hemopoiesis, or it may appear as a post-transplant complication in the new hemopoietic environment [62, 65–67]. CHIP has been associated with a higher risk for GvHD, as well as accelerated epigenetic aging [66, 68]. Future studies might examine if CHIP is involved in the interplay between hemostasis and complement.

### Diagnosis and treatment of macro-thrombotic events

The diagnosis of thrombotic events in HCT patients is challenging, as clinical signs are often unspecific and overlap with other complications. The use of biomarkers such as D-dimer and prothrombin fragment 1 + 2 is hampered by very low specificity and sensitivity and can be affected by other factors, such as inflammation, infection, and liver dysfunction. Other common prediction scores for venous thromboembolism (VTE), such as the Vienna prediction score [69] or the DASH score [70], cannot be applied in the context of HCT because of the substantial changes in cellularity and low specificity of biomarkers. In addition, quantitative changes in coagulation enzymes and natural inhibitors cannot fully explain the occurrence of peri-transplant thrombosis. However, the simultaneous occurrence of multiple concomitant risk factors seems to be relevant. Prevention and treatment of thrombotic events in HCT settings are based on benefit–risk considerations of anticoagulation therapy. Prophylactic anticoagulation with heparins can reduce the incidence of VTE in HCT but may also increase the risk of bleeding, especially in patients with thrombocytopenia, mucositis, or liver dysfunction [52, 71]. The use of fully dosed anticoagulation at therapeutic levels with warfarin, direct oral anticoagulants, or heparins can provide treatment for evident thrombotic events in HCT but may be complicated by drug interactions, adverse effects, and monitoring challenges in these patients. The optimal duration, dose, and type of anticoagulation in the peri-transplant setting are not well established and remain a matter of discussion relating to individual risk–benefit assessment and clinical responses.

## Focus on microangiopathic thrombosis as a dysregulation of hemostasis and complement

The complement system plays a crucial role in HCT, as it may be involved in beneficial and detrimental effects on transplant outcomes [72, 73]. On the one hand, the complement system can facilitate the engraftment of donor stem cells, promote graft-versus-leukemia effect, and contribute to protection against infections [72, 74–77]. On the other hand, the complement system can also be involved in the development of HCT-related complications in patients [72] and mouse models, such as GvHD [78, 79], transplant-associated thrombotic microangiopathy (HSCT-TMA) [29], sinusoidal obstruction syndrome/veno-occlusive disease (SOS/VOD), and infectious complications [72]. Due to dysregulated complement and hemostasis, endothelial activation and endothelial cell injury play important roles in the pathogenesis of these life-threatening complications after allogeneic HCT.

HSCT-TMA is characterized by microangiopathic hemolytic anemia, thrombocytopenia, and renal or neurologic complications in a post-HCT setting [80–83] and is more prevalent in patients undergoing allogeneic HCT. However, it has also been described in autologous HCT, mainly in pediatric patients [84]. Reported incidences of TMA after HCT are variable, ranging from 5.6–44.0%. Cytopenias and organ dysfunction, common events in HSCT recipients, impede the prompt diagnosis of the syndrome, and alternative causes for renal and neurologic dysfunction post-HCT might be at play [85, 86]. HSCT-TMA has been recognized as an actual “endothelial injury syndrome”. Several factors, including the toxicity of conditioning, the administration of calcineurin inhibitors, alloreactivity, bacterial products, and GvHD play a role in the development of a prothrombotic state that may contribute to the pathogenesis of thrombotic events in the microvasculature [87]. Thus, thrombosis in patients with HSCT-TMA often results from the interaction of complement, the coagulation cascade, and neutrophils [88]. In contrast to thrombotic thrombocytopenic purpura (TTP), ADAMTS 13 is not deficient in this syndrome and is considered inadequate as a disease marker [89, 90] consistent with plasma exchange not being convincingly effective for the management of HSCT-TMA in several cohorts [91]. Complement activation might play an important role in HSCT-TMA [92, 93]. In vitro activation of the complement system has been shown in small studies of pediatric patients with HSCT-TMA. Mezö et al. introduced soluble C5b-9 as a predictive marker of the later development of TMA in allogeneic HCT recipients [94]. Rotz et al. reported increased complement activation in a small cohort of HCT-TMA patients, using the modified Ham (mHAM) test, which was originally described for atypical hemolytic uremic syndrome [95]. Jodele et al. first described mutations in complement-related genes in pediatric patients after HCT [93], as well as an adverse prognostic effect of mutations in the alternative pathway of complement (APC)-related genes [96]. Data about genetic susceptibility at the complement factor and ADAMTS13 levels have also been reported in adult transplant settings. Like in aHUS, a two-hit pathogenesis might also play a role in HSCT-TMA. The first hit involves the genetic susceptibility of complement activation in these patients, while as a second hit, clinical factors may be implicated, such as donor type, age, conditioning, calcineurin or mTOR inhibitors, GVHD, or infections. Moreover, thrombin generation in patients receiving anti-thymocyte globulins (ATG) has also been associated with excess complement activation.

Various diagnostic criteria are available for HSCT-TMA. The Blood and Marrow Transplant Clinical Trials Network (BMT-CTN) and International Working Group (IWG) criteria have been widely used. In comparison to the BMT-CTN criteria, in the IWG criteria for the diagnosis of HSCT-TMA, organ damage is not needed, while a wider spectrum of patients with microangiopathic hemolytic anemia that may not be a result of complement system activation are included [97]. The diagnostic criteria of the TMA Harmonization Panel are the most up-to-date criteria proposed for the diagnosis of HSCT-TMA, including both biopsy-proven disease from the kidney or gastrointestinal system and clinical criteria [98]. Increased serum levels of neutrophil extracellular traps (NETs) have been recognized as predictors of HSCT-TMA onset in adult and pediatric populations [88, 99]. Despite substantial efforts to define soluble biomarkers of endothelial activation after HCT as predictors for TMA, no such biomarkers have been established [49, 100, 101].

GVHD is the main cause of mortality after allogeneic HCT in the absence of relapse or secondary malignancy [33, 34, 102]. Complement dysregulation has been implicated in GVHD in murine systems and humans [30, 103]. Inhibition of the alternative pathway (APC) by compstatin, a C3-targeted complement inhibitor, has led to reduced proliferation of T cells and Th1/Th17 polarization, as shown in human cutaneous tissues [77, 104]. Recently, the C5a/C5aR IL-17A axis was implicated in the development of chronic GVHD in an in vitro study [105]. Levels of C3 have been correlated with sclerotic cutaneous GVHD, while patients with these lesions have abnormalities in complement factor H and APC functional assays [72, 106]. In addition, findings on intracellular components of the complement system acting in parallel to the humoral model might provide insights into the pathogenesis of endothelial injury in GVHD [16]. One might speculate that T cells containing C3 may mediate complement activation and inflammation on and/or underneath the endothelial cell layer after migration, thus promoting endothelial injury as a component of GVHD pathogenesis [16, 107]. Markers of endothelial dysfunction, such as endothelial microvesicles, are increased 2-3 weeks after allogeneic HSCT and in patients with acute GVHD [108, 109]. Treatment of GVHD with complement inhibitors is still under investigation. One prospective phase 2a study has examined the role of complement inhibition on adult patients with acute GVHD (aGVHD) and involvement of the lower gastrointestinal tract [77]. The researchers examined the efficacy of ALXN1007 (C5a inhibitor) administration in combination with corticosteroids in a cohort of 25 patients. The overall response rate 28 days post-treatment initiation was 58%.

SOS/VOD is considered a rare but severe complication of allogeneic HCT [31] with increased risk upon application of gemtuzumab or inotuzumab prior to HCT or in preexisting liver damage [110, 111]. SOS/VOD pathogenesis is correlated with damage to sinusoidal endothelial cells and hepatocytes that contributes to venular occlusion, and modifiable vs. persistent risk factors for SOS/VOD have been described in detail [31]. Little is known about changes in complement parameters during SOS/VOD. Since SOS/VOD is related to endothelial damage, complement activation might find a suitable substrate to develop, in analogy to the development of HELLP-type microangiopathies during pregnancy [112–114]. Various biomarkers have been reported as predictive tools, but none have been reliably established for routine clinical application [31, 49].

## Treatment approaches for HSCT-TMA addressing dysregulated hemostasis and complement

Increasing insights into the underlying mechanisms of HSCT-TMA have revolutionized the management of affected patients. Complement inhibition constitutes a cornerstone in the therapeutic approach to HSCT-TMA and has proven effective for patients with other TMAs, such as atypical hemolytic uremic syndrome [115, 116]. Eculizumab, a C5 inhibitor, inhibits the terminal pathway of the complement system and has been widely used in HSCT-TMA [117, 118]. Real-world data have shown that early administration of eculizumab in patients with increased activation of the complement system, as assessed by measurement of C5b-9 levels, close monitoring of treatment response, and dose modification, when necessary, results in better outcomes [119]. Before the era of therapeutic complement inhibition, the management of HSCT-TMA included supportive treatment, calcineurin or mammalian target of rapamycin (mTOR) inhibitors, and corticosteroids. Moreover, plasma exchange or infusions of rituximab were also used, according to each center’s policy. Eculizumab has revolutionized the management of both pediatric and adult patients with HSCT-TMA [117–119]. In some studies, the response rate to eculizumab reached 93%. However, the overall survival of patients with HSCT-TMA remains low at approximately 30% [118], while Jodele et al. reported higher 1-year survival rates of 66% in a cohort of pediatric eculizumab-treated patients vs. 17% in a historical control group [119]. Table 1 illustrates the published studies on the use of complement inhibitors in HSCT-TMA [117, 119–127]. These agents have not yet been officially approved for use in these patients.

**Table 1:** Studies examining the role of complement inhibitors in the management of HSCT-TMA, having not received yet official approval for administration in these patients (HSCT: hematopoietic stem cell transplantation, HSCT-TMA: hematopoietic stem cell transplantation-associated thrombotic microangiopathy, PE: Plasma exchange therapy).

First author, Year of publication (ref)	Complement inhibitor	Study design	Study population	Prior HSCT-TMA treatments	Comparator	Outcomes
Jodele, 2014 (120) PMID: 24370861	Eculizumab	Monocenter, retrospective study	6 pediatric patients	5 patients received PE	–	HSCT-TMA resolved in 4 of 6 children
Fontbrune, 2015 (121) PMID: 25651309	Eculizumab	Multi-center, retrospective study	12 patients (3 children and 9 adults)	7 patients received prior treatment before Eculizumab (PE in 7 patients and rituximab in two patients)	–	In 14-month follow-up: 50% hematologic response 33% overall survival Were reported
Vasu, 2016 (117) PMID: 27064689	Eculizumab	Monocenter, retrospective study	5 adult patients	–	–	4 out of 5 responded to Eculizumab treatment
Rudoni, 2018 (123) PMID: 29920784	Eculizumab	Monocenter, retrospective study	10 adult patients	In 3 patients Eculizumab was second line treatment (intravenous immune globulin, steroids, PE, discontinuation of immunosuppressive agents)	–	Overall hematologic response rate 70% Overall survival rate 60%
Jan, 2019 (122) PMID: 31587288	Eculizumab	Monocenter, comparative retrospective study	10 adult patients	In 3 patients Eculizumab was second line treatment (PE, supportive care, discontinuation of immunosuppressive agents)	10 patients who received conventional treatment	7 patients in the eculizumab group achieved hematologic response (1 patient achieved complete response with organ recovery) Overall survival was 60% in the eculizumab cohort and 30% in the conventional cohort.
Jodele, 2020 (124) PMID: 31932840	Eculizumab	Multi-center, retrospective study	64 pediatric patients	–	An untreated cohort with same HSCT-TMA characteristics	66% improvement in 1-year post-HSCT survival
Khaled, 2022 (125) PMID: 35439028	Narsoplimab	Phase II, single-arm, study	28 adult patients	–	–	Response rate 61% (laboratory HSCT-TMA markers)
Svec, 2023 (126) PMID: 36333550	Eculizumab	Multi-center, retrospective study	82 pediatric patients	–	–	The cumulative incidence of HSCT-TMA resolution was 36.6% The overall survival (OS) was 47.1%
Jodele, 2024 (127) PMID: 37946262	Eculizumab	Multi-center, prospective study	21 pediatric and young adult patients	–	–	Survival of 71% 6 months after HSCT-TMA diagnosis In 73% of patients fully recovered organ function was reported
Benítez Carabante, 2024 (128) PMID: 38521410	Eculizumab	Multi-center, retrospective study	29 pediatric patients	–	–	65.5% of patients responded to eculizumab (58.6% achieved a complete response and 6.9% achieved a partial response)

**Fig. 3:**
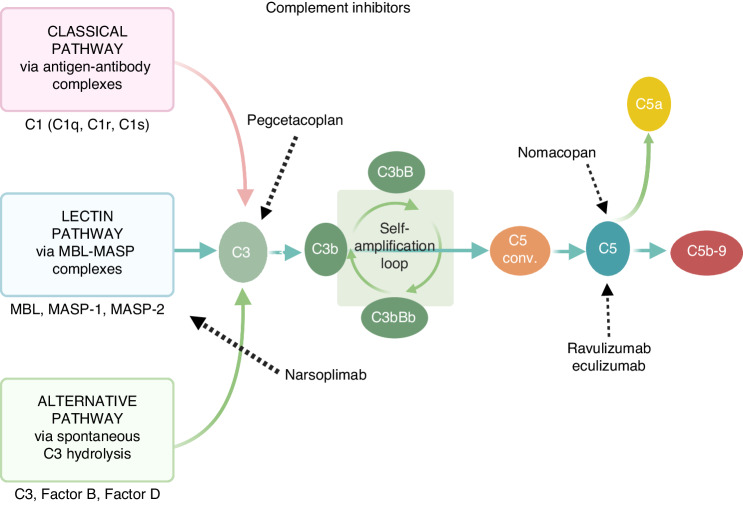
Simplified scheme of the complement cascade and mode of action of drugs in clinical use or development as complement inhibitors in the HSCT setting (MASP-2: mannose-binding protein-associated serine protease-2, C1-C3-C5: Complement factors, conv.: convertase) [16].

Next-generation complement inhibitors are currently under investigation for the treatment of adult and pediatric patients with HSCT-TMA. A phase 2 single-arm, open-label study of narsoplimab (a MASP-2 inhibitor) in 19 HSCT-TMA patients reported a greater median OS than in a historical control group [124]. This drug is currently under priority review by the FDA. Ravulizumab, a C5 inhibitor approved for the treatment of paroxysmal nocturnal hemoglobinuria, aHUS, generalized myasthenia gravis, and neuromyelitis optica spectrum disorder, is being investigated in a phase 3 trial in adults (NCT04543591) and children (NCT04557735). Coversin or nomacopan, a C5 inhibitor that blocks leukotriene B4, is under investigation in a two-part phase 3 trial of pediatric HSCT-TMA (NCT04784455). Complement inhibitors in use or under study as therapeutics for the management of HSCT-TMA are presented in Table 2 and Fig. 3 [16].

**Table 2:** Next-generation complement therapeutics in use and under investigation in HSCT-TMA [16].

Agent	Target	Development for HSCT-TMA
**Pegcetacoplan** (APL-2)	C3	Phase 2 Early access program
**Narsoplimab** (OMS721)	MASP-2	Phase 3; Expanded access program
**Ravulizumab** (ALX1210)	C5	Phase 3
**Nomacopan** (Coversin)	C5 and LTB4 inhibition	Phase 3 (pediatric HSCT-TMA)

*MASP-2* mannose-binding protein-associated serine protease-2, *HSCT-TMA* transplant-associated thrombotic microangiopathy, *LTB4* leukotriene B4, *C3-C5* complement factors.

Since inflammation is a common denominator for thrombotic events, triggered by hemostasis and/or complement activation, interventions that reduce or restrict inflammation might prove beneficial [128]. Newly developed targeted coagulation factor XI inhibitors, used as antithrombotics, have demonstrated clear anti-inflammatory activity as a surrogate effect in humans and in experimental studies [129, 130]. Currently, they are being tested as anticoagulants only in phase II and III trials in patients with stroke and in patients with atrial fibrillation as protection against stroke [131, 132]. One might speculate that factor XI inhibitors might prove more efficient than their competitors if given as thromboprophylaxis in patients with HCT [133, 134]. From the physiology point of view activation of the contact phase of coagulation can trigger hemostasis but dispense unexpected thrombus formation [132]. They seem promising in patients with severe kidney disease or cancer-associated thrombosis, an area where other established direct oral anticoagulants are excluded. The anti-inflammatory action of FXI-inhibitors might prove beneficial against complement activation, or they might act synergistically with complement activation inhibitors [24]. Major drawbacks for their use, though, might be their known potentially unfavorable drug-drug interactions and, until present, the lack of direct antidotes for the case of a bleeding complication [135].

## Future research and clinical prospects

Thrombotic events are increasingly recognized as a distinct clinical complication in the context of HCT. Investigating the pathophysiology of thrombosis becomes more complex as the function and interplay of multiple effectors, such as Hemostasis and Complement, are further elucidated. Adverse reactions of mechanisms of the innate immunity resulting in thrombotic events were only recently described as the concept of Immunothrombosis [136–138]. They involve the role and interaction of various components, such as innate immune cells, platelets and coagulation factors, in an effort to clear local infections. The immune system in both its humoral and cellular dimension is strongly challenged and affected during the process of HCT. Thrombotic events have a variable impact on the immune system triggering inflammatory responses, an observation known as thromboinflammation [136]. Understanding thromboinflammation and immunothrombosis, through the dual role of Hemostasis and Complement as biological defense systems, helps explain micro- or macro-thrombotic complications in HCT and design better treatment approaches. Iatrogenic interventions during the process of HCT could also be added to the list of triggering mechanisms for thrombotic events, as the catheter related thrombosis clearly demonstrates.

Given the high mortality and morbidity that patients with HSCT-TMA experience, identification of novel biomarkers aiming for early diagnosis of this severe disease entity is crucial for better outcomes for our patients. Future research efforts should focus on the role of next-generation complement therapeutics in the management of this distinct type of TMA, examining not only their impact on the overall survival but also on the quality of life of the patients. Genetic and functional data regarding complement activation in patients with GVHD are crucial, while research should investigate the role of alternative and lectin pathways in the pathogenesis of this syndrome. Moreover, multi-center collaboration can be helpful for the examination of the safety and efficacy of complement inhibitors in aGVHD. SOS/VOD constitutes a rare but fatal HSCT complication. The genetic background of SOS/VOD should also be investigated, contributing to the development of targeted therapies.
